# Clinical Data based XGBoost Algorithm for infection risk prediction of patients with decompensated cirrhosis: a 10-year (2012–2021) Multicenter Retrospective Case-control study

**DOI:** 10.1186/s12876-023-02949-3

**Published:** 2023-09-13

**Authors:** Jing Zheng, Jianjun Li, Zhengyu Zhang, Yue Yu, Juntao Tan, Yunyu Liu, Jun Gong, Tingting Wang, Xiaoxin Wu, Zihao Guo

**Affiliations:** 1 Operation Management Office, Affiliated Banan Hospital of Chongqing Medical University, Chongqing, 401320 China; 2 Department of Cardiothoracic Surgery, Affiliated Banan Hospital of Chongqing Medical University, Chongqing, 401320 China; 3 Medical Records Department, the First Affiliated Hospital, Zhejiang University School of Medicine, Hangzhou, 310003 China; 4 Senior Bioinformatician Department of Quantitative Health Sciences, Mayo Clinic, Rochester, MN 55905 US; 5 Medical Records Department, the Second Affiliated Hospital of Chongqing Medical University, Chongqing, 400010 China; 6 Department of Information Center, the University Town Hospital of Chongqing Medical University, Chongqing, 401331 China; 7 College of Medical Informatics, Chongqing Medical University, Chongqing, 400016 China; 8 State Key Laboratory for Diagnosis and Treatment of Infectious Diseases, National Clinical Research Centre for Infectious Diseases, the First Affiliated Hospital, Zhejiang University School of Medicine, 79 Qing Chun Road, Hangzhou, 310003 Zhejiang China; 9 Department of Gastroenterology, Chongqing Banan Cancer Hospital, Chongqing, 400054 China

**Keywords:** Decompensated cirrhosis, Infection, XGBoost algorithm, Prediction model, Multicenter

## Abstract

**Objectives:**

To appraise effective predictors for infection in patients with decompensated cirrhosis (DC) by using XGBoost algorithm in a retrospective case-control study.

**Methods:**

Clinical data were retrospectively collected from 6,648 patients with DC admitted to five tertiary hospitals. Indicators with significant differences were determined by univariate analysis and least absolute contraction and selection operator (LASSO) regression. Further multi-tree extreme gradient boosting (XGBoost) machine learning-based model was used to rank importance of features selected from LASSO and subsequently constructed infection risk prediction model with simple-tree XGBoost model. Finally, the simple-tree XGBoost model is compared with the traditional logical regression (LR) model. Performances of models were evaluated by area under the receiver operating characteristic curve (AUROC), sensitivity, and specificity.

**Results:**

Six features, including total bilirubin, blood sodium, albumin, prothrombin activity, white blood cell count, and neutrophils to lymphocytes ratio were selected as predictors for infection in patients with DC. Simple-tree XGBoost model conducted by these features can predict infection risk accurately with an AUROC of 0.971, sensitivity of 0.915, and specificity of 0.900 in training set. The performance of simple-tree XGBoost model is better than that of traditional LR model in training set, internal verification set, and external feature set (P < 0.001).

**Conclusions:**

The simple-tree XGBoost predictive model developed based on a minimal amount of clinical data available to DC patients with restricted medical resources could help primary healthcare practitioners promptly identify potential infection.

**Supplementary Information:**

The online version contains supplementary material available at 10.1186/s12876-023-02949-3.

## Introduction

The natural history of cirrhosis is characterized by an asymptomatic compensated phase followed by a decompensated phase, marked by the development of overt clinical signs, the most frequent of which are ascites, bleeding, encephalopathy, and jaundice [1–3]. Patients with decompensated cirrhosis (DC) are critically ill with high mortality. A study has shown that, compared with compensated cirrhosis, the annual mortality rate of patients with DC reaches 20%, which is much higher than the 7% of patients with compensated cirrhosis [4]. At the same time, patients with DC have more complications, and infection is the most common complication [5]. There are many kinds of infection caused by cirrhosis, such as spontaneous bacterial peritonitis (SBP) [6, 7], urinary system infection [8], and spontaneous bacteremia [9, 10]. Infection is also an important inducing factor of severe complications such as upper gastrointestinal bleeding, hepatic encephalopathy, and hepatorenal syndrome, and is one of the main causes of death of patients with advanced liver cirrhosis [11–13]. Over the past few decades, various cohort studies have evaluated SBP-related in-hospital mortality. From December 1984 to February 1989, the Liver Unit at the University of Barcelona Hospital Clinic reported a 38% in-hospital mortality in 185 consecutive cirrhotic patients with SBP [14]. In another 10-year cohort study (from 1988 to 1998), Maryland hospitals reported that 112 of 343 patients with SBP died in the hospital, with a mortality rate of 32.6% [15]. Thus, patients with DC complicated with infection usually have a poor prognosis. Therefore, identifying the risk factors of DC complicated with infection and constructing the prediction model are of great significance for improving the prognosis quality and reducing the risk of mortality in DC Patients.

As an artificial intelligence, machine learning algorithm has been applied in the field of disease prediction and diagnosis [16–18]. Classical machine learning algorithms and models include decision tree model and integration tree model, among which support vector machines (SVM) [19] and neural network models (NNs) [20] are more commonly used, while XGboost is the most commonly used integration tree algorithm [21]. Among many machine learning algorithms and models, logistic regression (LR) is more suitable for processing linear variables, while XGboost, multilayer perceptron (MLP), random forest (RF), naive bayes (NB) and SVM have strong nonlinear variable processing capabilities [22–24]. In addition, XGboost has become one of the most successful algorithms in machine learning competitions, and has been widely used and achieved good results.

Kim et al. developed 55 machine learning models (RF, NNs, XGBoost, generalized linear model, etc.) to predict the needs of patients with COVID-19 for intensive care, and found that XGBoost model showed the highest recognition performance. The area under the receiver operating characteristic curve (AUROC) of XGBoost model in the development group is 0.897, and that in the validation group is 0.885. This model can effectively predict the demand for intensive care of patients with COVID-19 [25]. Huang et al. used the traditional Cox proportional risk model and three machine learning models to construct and screen the best recurrence prediction model after resection of hepatocellular carcinoma for early monitoring and identification of high-risk patients with recurrence. The results showed that in the internal validation set, XGBoost model obtained the best discrimination with a C index of 0.713, which affirmed the value and role of XGBoost model in prediction [26].

Although the importance of XGBoost in clinical decision-making has been gradually recognized by clinicians. However, its value in predicting infection in patients with DC has not been reported. Therefore, we designed this study to develop an XGBoost model combining demographic characteristics, etiology, complications, and laboratory indicators to predict the risk probability of infection in patients with DC, and further compared the value of the XGBoost model with the prediction method based on the conventional LR.

## Methods

### Study design and patients

Clinical data of this study were obtained from five third-level hospitals in southwest China. In this multicenter retrospective study, 6,648 of 10,689 DC patients with clinical consultation records met the quality standards for the final analysis. These patients were randomly divided into a training set with 4,353 samples and an internal validation set with 1,866 samples from hospitals A-D at a ratio of 7:3. A total of 429 samples from hospital E were used for external validation. The study adhered to the principles of the Declaration of Helsinki and the Transparent Reporting of a Multivariable Prediction Model for Individual Prognosis or Diagnosis Guidelines [27]. Clinical research ethics approval was obtained from the Ethics Committee of the Affiliated Banan Hospital of Chongqing Medical University (approval number: 2021-008). Individual patient-level consent was not required because the study only used fully de-identified collected data.

### Diagnostic criteria

The diagnosis of DC is confirmed by liverbiopsy, clinical, biochemical, and imaging data or past medical records, and the diagnosis is in accordance with the “EASL Clinical Practice Guidelines for the management of patients with decompensated cirrhosis” [1]. Infection was defined to include SBP, pneumonia, cellulitis, urinary system infection and spontaneous bacteremia, and (ii) a combination of microbial detection, clinical or laboratory signs of infection [28, 29].

### Inclusion and exclusion criteria

The inclusion criteria for this study were DC patients admitted between July 2012 and December 2021. Exclusion criteria were as follows: (i) age < 18 years, (ii) patients with cancer other than primary liver cancer, (iii) mental illness, (iv) pregnant and lactating women, and (v) variables with > 30% missing values. The detailed selection process is shown in Supplementary Fig. 1.

### Data collection

On the basis of previous studies, 28 variables routinely tested or recorded were collected, which included age, sex, hypertension, diabetes, smoking, drinking, primary liver cancer, family history of liver disease, hepatitis B virus (HBV), hepatitis C virus (HCV), alcoholic, autoimmunity, gastrointestinal bleeding (GIB), ascites, hepatic encephalopathy (HE), hepatic failure (HF), total protein (TP), total bilirubin (TB), hemoglobin, blood sodium (Na), blood potassium (K), albumin (ALB), prothrombin activity (PTA), blood urea nitrogen (BUN), creatinine (Cr), red blood cell (RBC) count, white blood cell (WBC) count, and neutrophils to lymphocytes ratio (NLR). Considering that many features may have different values when measured at different time points, we only included the first measurement values of patients after their first admission in this study.

### Statistical analysis

Statistical analysis was performed using SPSS 22.0 and R software (version 4.0.2, Vienna, Austria). Kolmogorov Smirnov Normality test was applied for quantitative data. Probability (P) values of > 0.05 were considered normal distribution. The data with a normal distribution were presented as the mean ± standard deviation and tested with t-test, whereas those with a non-normal distribution were described with the median (interquartile range, [IQR]) and tested with Mann-Whitney U test. The qualitative data were presented as n (%) and tested with χ^2^ test. We used the R multivariate imputation by chained equation package for missing data imputation in this study.

In the model construction phase, we developed the LR and XGBoost algorithm models. First, the variables with statistical differences were identified through single factor analysis. Then the least absolute shrinkage and selection operator (LASSO) regression was used to further screen potential related variables. Finally, LR and XGBoost models were constructed to analyze the impact of each variable on the increased risk of infection in patients with DC. The hyperparameters of XGBoost were set as follows: eta = 0.3, max_depth = 5, subsample = 0.5, colsample_bytree = 1, gamma = 0.5. We defined this model as “multi-tree XGBoost” and the ranks of feature importance were then obtained [30]. The correlation between the multi-tree XGBoost model’s features was evaluated using Pearson correlation analysis. In order to further determine the most significant features related to infection risk in the unbalanced data, we conducted 100-round 5-fold cross-validation in the training set. When the seventh feature was added in the XGBoost model, the increased AUROC was less than 0.5% (P = 0.158, Supplementary Fig. 2). Finally, six features were selected as significant predictors and defined the model as “simple-tree XGBoost”.

All statistical analyses were two-sided, and statistical significance was set at P < 0.05. Moreover, the “rms”, “ggplot2”, “glment”, “plotROC”, “reportROC”, “corrplot”, “caret”, “dplyr”, and “XGBoost” packages in R were used in our study.

## Results

### Patient characteristics

The Mann-Whitney U test revealed that there was no significant difference in all missing variables in the training and internal validation sets before and after multiple imputations (Supplementary Table 1). Furthermore, there were no significant differences in all missing variables in the external validation set before and after multiple imputations (Supplementary Table 2). Table 1 summarizes the clinical characteristics of patients in the training and internal validation sets. No significant differences were observed in any of the variables between the two groups (P > 0.05). Patients in the training set were divided into infection and non-infection groups. Univariate analysis revealed that the following variables were significantly associated with infection: sex, hypertension, diabetes, smoking, drinking, primary liver cancer, alcoholic, autoimmunity, GIB, HE, HF, TP, TB, hemoglobin, Na, K, ALB, PTA, BUN, Cr, RBC count, WBC count, and NLR **(**Table 2**)**.

**Table 1:** Demographic and clinical characteristics of the training and internal validation sets

Variables	Total (N = 6219)	Training set (N = 4353)	Internal validation set (N = 1866)	P value
Age	56.00(49.00,66.00)	56.00(49.00,67.00)	56.00(49.00,66.00)	0.631
Sex	4491(72.21)	3165(72.71)	1326(71.06)	0.194
Hypertension	712(11.45)	497(11.42)	215(11.52)	0.940
Diabetes	1011(16.26)	722(16.59)	289(15.49)	0.299
Smoking	2901(46.65)	2057(47.25)	844(45.23)	0.150
Drinking	2844(45.73)	1997(45.88)	847(45.39)	0.746
Primary liver cancer	670(10.77)	474(10.89)	196(10.50)	0.686
Family history of liver disease	1377(22.14)	944(21.69)	433(23.20)	0.198
HBV	4028(64.77)	2798(64.28)	1230(65.92)	0.226
HCV	252(4.05)	184(4.23)	68(3.64)	0.318
Alcoholic	883(14.20)	614(14.11)	269(14.42)	0.778
Autoimmunity	480(7.72)	339(7.79)	141(7.56)	0.794
GIB	1128(18.14)	798(18.33)	330(17.68)	0.568
Ascites	240(3.86)	164(3.77)	76(4.07)	0.616
HE	375(6.03)	266(6.11)	109(5.84)	0.726
HF	863(13.88)	623(14.31)	240(12.86)	0.140
TP (IQR, g/L)	64.70(58.60,71.60)	64.70(58.60,71.70)	65.00(58.70,71.30)	0.651
TB (IQR, umol/L)	29.60(17.40,71.70)	29.60(17.30,71.80)	29.90(17.80,71.45)	0.993
Hemoglobin (IQR, g/L)	108.00(85.00,127.00)	108.00(84.00,127.00)	109.00(86.00,127.00)	0.505
Na (IQR, mmol/L)	139.30(136.20,141.70)	139.30(136.20,141.70)	139.20(136.20,141.60)	0.404
K (IQR, mmol/L)	3.87(3.56,4.19)	3.87(3.55,4.19)	3.85(3.57,4.18)	0.741
ALB (IQR, g/L)	31.20(27.20,35.40)	31.20(27.30,35.40)	31.20(27.10,35.40)	0.500
PTA (IQR, %)	64.00(49.00,78.00)	63.60(49.00,78.00)	64.00(49.00,79.00)	0.329
BUN (IQR, mmol/L)	5.40(4.12,7.39)	5.40(4.11,7.36)	5.41(4.16,7.41)	0.982
Cr (IQR, umol/L)	67.00(56.00,81.70)	67.20(56.10,81.70)	66.70(56.00,81.80)	0.763
RBC count (IQR, ×10^9^/L)	3.52(2.89,4.12)	3.51(2.88,4.11)	3.53(2.92,4.15)	0.267
WBC count (IQR, ×10^9^/L)	4.30(3.00,6.34)	4.32(2.99,6.35)	4.29(3.03,6.27)	0.876
NLR (IQR)	3.40(2.08,5.80)	3.37(2.08,5.82)	3.44(2.07,5.76)	0.916

HBV: hepatitis B virus; HCV: hepatitis C virus; GIB: gastrointestinal bleeding; HE: hepatic encephalopathy; HF: hepatic failure; TP: total protein; TB: total bilirubin; Na: blood sodium; K: blood potassium; ALB: albumin; PTA: prothrombin activity; BUN: blood urea nitrogen; Cr: creatinine; RBC: Red blood cell; WBC: white blood cell; NLR: neutrophils to lymphocytes ratio; IQR: interquartile range

**Table 2:** Univariate analysis of variables associated with infection

Variables	Training set	Infection group	Non-infection group	P value
	(N = 4353)	(N = 2266)	(N = 2087)	
Age	56.00(49.00,67.00)	56.00(49.00,66.00)	56.00(50.00,67.00)	0.544
Sex	3165(72.71)	1710(75.46)	1455(69.72)	< 0.001
Hypertension	497(11.42)	241(10.64)	256(12.27)	0.100
Diabetes	722(16.59)	412(18.18)	310(14.85)	0.004
Smoking	2057(47.25)	1116(49.25)	941(45.09)	0.007
Drinking	1997(45.88)	1107(48.85)	890(42.64)	< 0.001
Primary liver cancer	474(10.89)	217(9.58)	257(12.31)	0.004
Family history of liver disease	944(21.69)	497(21.93)	447(21.42)	0.708
HBV	2798(64.28)	1476(65.14)	1322(63.34)	0.230
HCV	184(4.23)	83(3.66)	101(4.84)	0.064
Alcoholic	614(14.11)	369(16.28)	245(11.74)	< 0.001
Autoimmunity	339(7.79)	149(6.58)	190(9.10)	0.002
GIB	798(18.33)	520(22.95)	278(13.32)	< 0.001
Ascites	164(3.77)	84(3.71)	80(3.83)	0.890
HE	266(6.11)	212(9.36)	54(2.59)	< 0.001
HF	623(14.31)	550(24.27)	73(3.50)	< 0.001
TP (IQR, g/L)	64.70(58.60,71.70)	62.20(56.50,68.30)	67.60(61.20,74.20)	< 0.001
TB (IQR, umol/L)	29.60(17.30,71.80)	46.20(21.60,164.40)	22.50(14.70,37.60)	< 0.001
Hemoglobin (IQR, g/L)	108.00(84.00,127.00)	103.00(81.00,123.00)	113.00(89.00,131.00)	< 0.001
Na (IQR, mmol/L)	139.30(136.20,141.70)	138.00(134.60,141.00)	140.20(138.00,142.20)	< 0.001
K (IQR, mmol/L)	3.87(3.55,4.19)	3.84(3.46,4.23)	3.90(3.61,4.14)	0.003
ALB (IQR, g/L)	31.20(27.30,35.40)	29.00(26.00,32.78)	33.70(29.90,38.00)	< 0.001
PTA (IQR, %)	63.60(49.00,78.00)	54.80(40.00,68.93)	72.00(60.00,85.00)	< 0.001
BUN (IQR, mmol/L)	5.40(4.11,7.36)	5.80(4.22,8.42)	5.13(4.03,6.66)	< 0.001
Cr (IQR, umol/L)	67.20(56.10,81.70)	69.00(57.43,87.10)	65.80(54.95,76.95)	< 0.001
RBC count (IQR, ×10^9^/L)	3.51(2.88,4.11)	3.32(2.73,3.96)	3.69(3.08,4.26)	< 0.001
WBC count (IQR, ×10^9^/L)	4.32(2.99,6.35)	5.20(3.43,7.95)	3.73(2.70,5.03)	< 0.001
NLR (IQR)	3.37(2.08,5.82)	4.94(2.96,7.95)	2.43(1.62,3.62)	< 0.001

HBV: hepatitis B virus; HCV: hepatitis C virus; GIB: gastrointestinal bleeding; HE: hepatic encephalopathy; HF: hepatic failure; TP: total protein; TB: total bilirubin; Na: blood sodium; K: blood potassium; ALB: albumin; PTA: prothrombin activity; BUN: blood urea nitrogen; Cr: creatinine; RBC: Red blood cell; WBC: white blood cell; NLR: neutrophils to lymphocytes ratio; IQR: interquartile range

### Clinical features selection in LASSO regression analysis

**Fig. 1:**
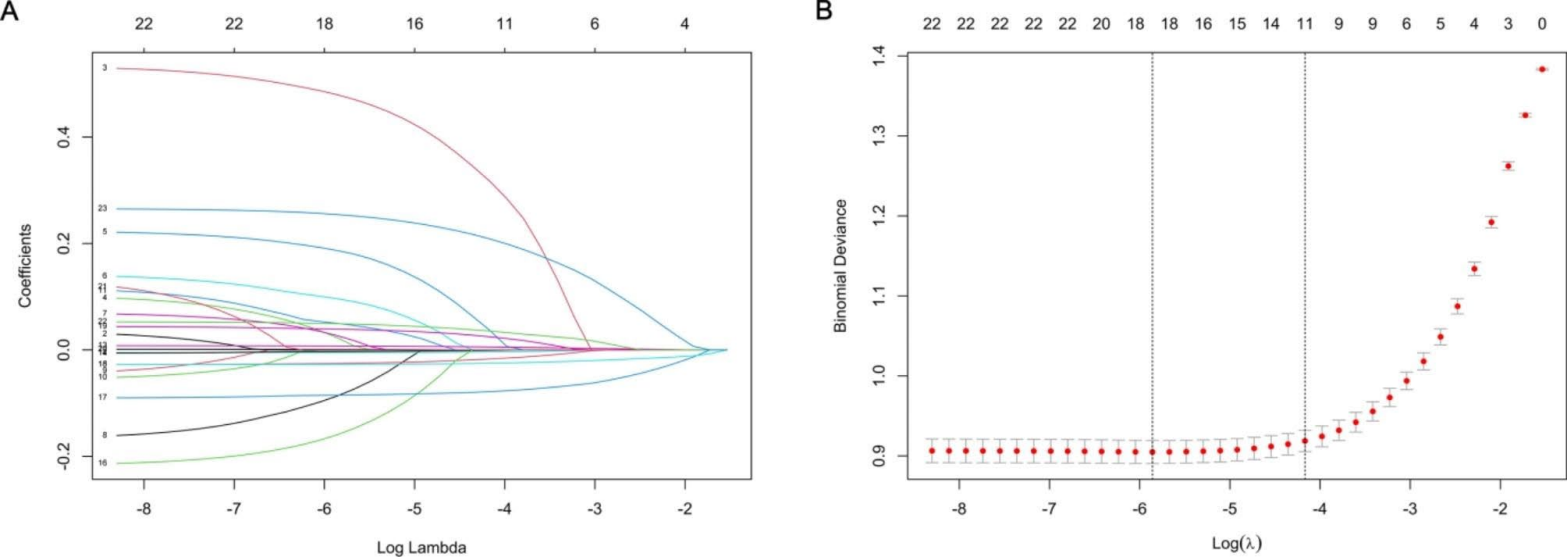
Features selection by LASSO. (A) LASSO coefficients profiles (y-axis) of the 22 features. The upper x-axis is the average numbers of predictors and the lower x-axis is the log(λ). (B) 10-fold cross-validation for tuning parameter selection in the LASSO model

Further, 22 features with statistical differences in univariate analysis were enter into the LASSO regression analysis, and 11 were significantly associated with infection, including GIB, HF, TP, TB, hemoglobin, Na, ALB, PTA, BUN, WBC count, and NLR **(**Fig. 1**)**.

**Fig. 2:**
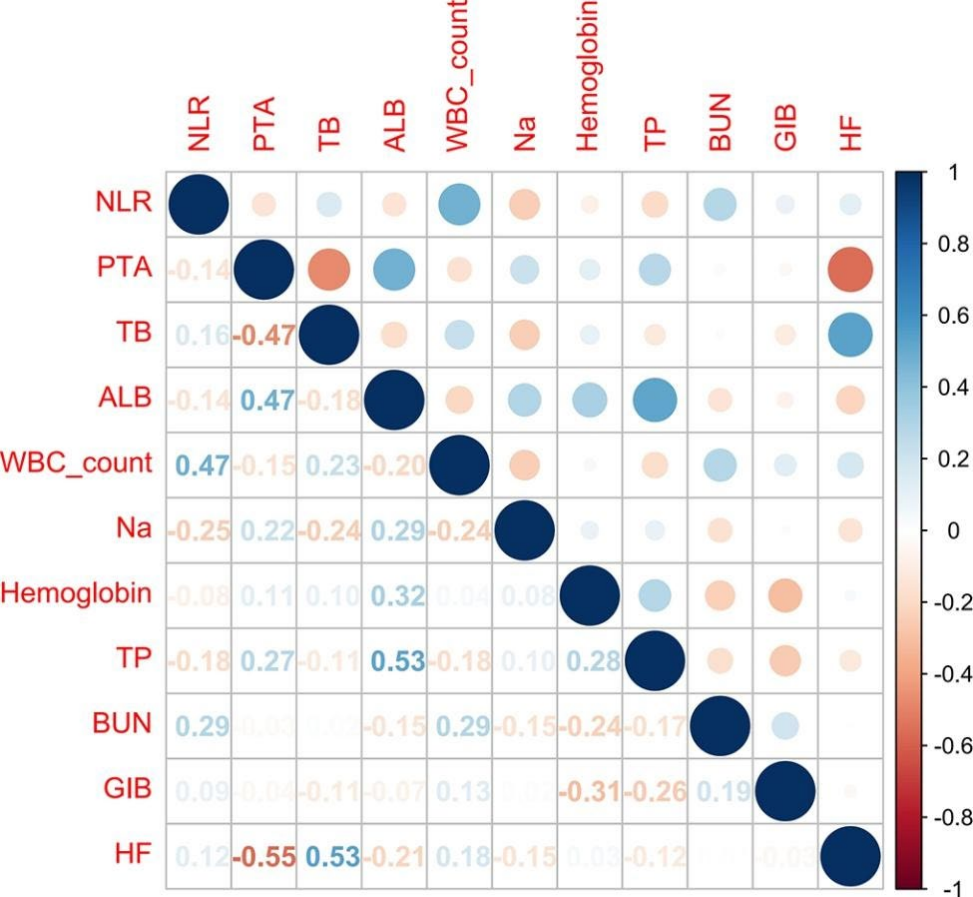
Correlation coefficient Matrices of 11 features

Figure 2 shows the correlation between these 11 features. There is a significant positive correlation between HF and TB (r = 0.53, P < 0.001), a significant positive correlation between TP and ALB (r = 0.53, P < 0.001), a significant negative correlation between HF and PTA (r=-0.55, P < 0.001), and a significant negative correlation between TB and PTA (r=-0.47, P < 0.001).

### Construction and evaluation of XGBoost model

**Fig. 3:**
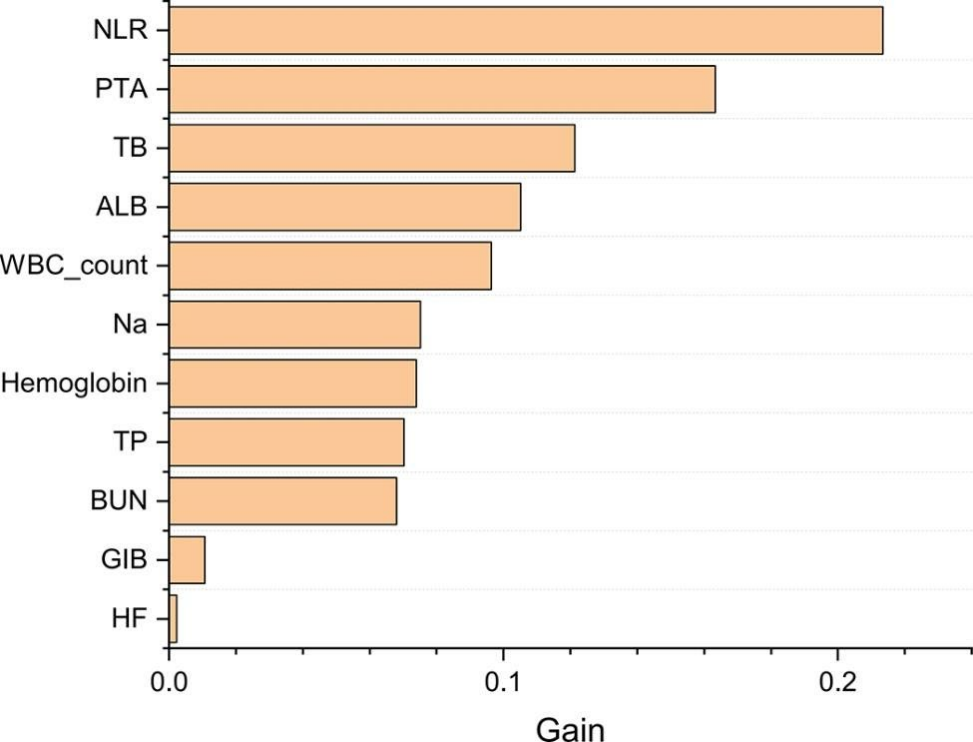
The rank of importance of 11 features in Mutil-tree XGBoost

The aforementioned 11 features were entered into multi-tree XGBoost. Figure 3 shown the rank of their importance. Subsequently, we added the ranked features one by one to the XGBoost model until an AUROC score improving inferior to 0.5%. Six features, including TB, Na, ALB, PTA, WBC count and NLR were selected as the significant factors. Then a simple-tree XGBoost model was constructed based on the above six key features.

**Fig. 4:**
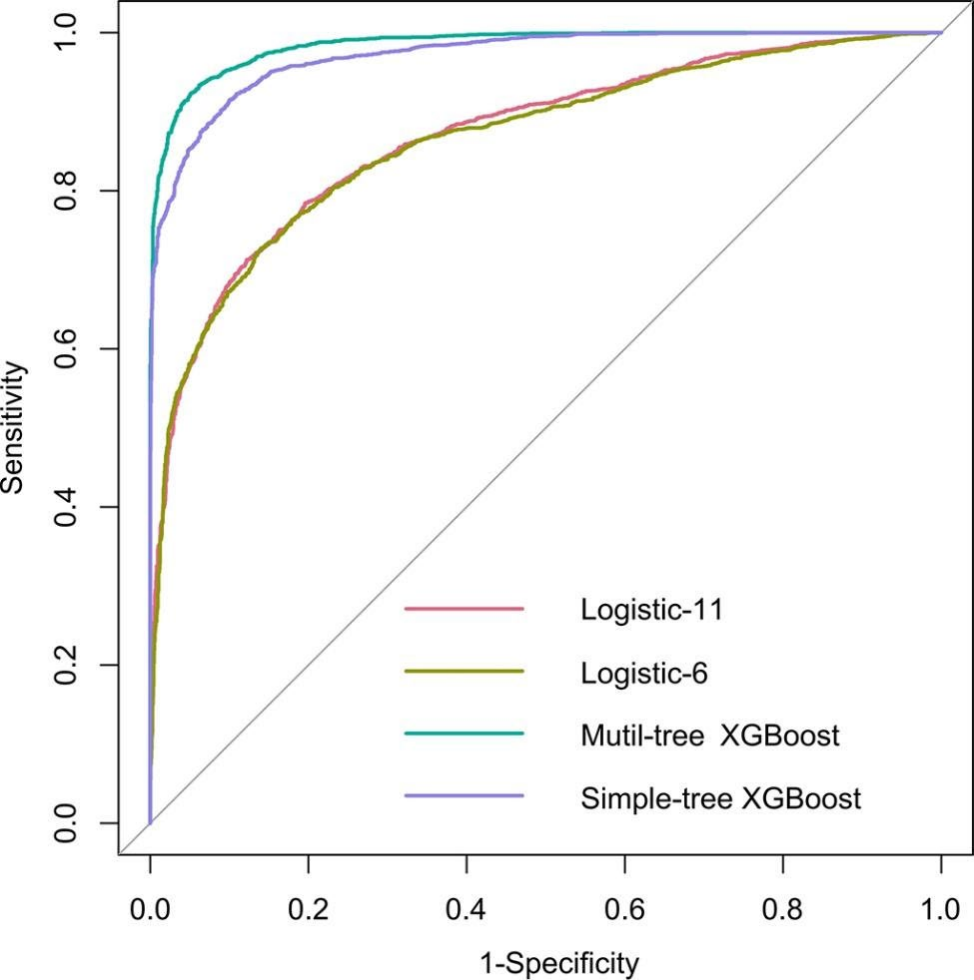
AUROC in training set

For the benchmark purpose, we also compared the performances of XGBoost model with the conventional multivariable LR model. In training set, the simple-tree XGBoost model with 6 selected features revealed superior performance compared to the LR with all 11 features (AUROC: 0.971 vs. 0.869, P < 0.001) or 6 features (AUROC: 0.971 vs. 0.864, P < 0.001) **(**Fig. 4**)**. Table 3 shown the detailed performance metrics for the four models in training set. We have provided the formula details of the performance criteria in Supplementary Table 3. Similarly, in internal validation set, the simple-tree XGBoost model exhibited better performance than the LR used by all 11 features (AUC: 0.998 vs. 0.878, P < 0.001) or the six selected features (AUC: 0.998 vs. 0.875, P < 0.001) (Supplementary Fig. 3). Supplementary Table 4 shown the detailed performance metrics for the four models in internal validation set. In the external validation set, the simple-tree XGBoost model by using six selected features and LR model by using 11 features showed a superior performance (AUC: 1.000 vs. 0.849, P < 0.001) (Supplementary Fig. 4). Supplementary Table 5 shown the detailed performance metrics for the four models in external validation set. Briefly, the above results suggested that simple-tree XGBoost model owned more precise and stable prediction performance than multivariable LR in identifying infection outcome of patients with DC. In addition, we have substituted patients from different centers into the model and compared the diagnostic agreement. The results showed no significant difference between the AUROC of each center and the AUROC of all centers (Supplementary Table 6).

**Table 3:** Detailed performance metrics for the four models in training set

Models	AUROC	Sensitivity	Specificity	PPV	NPV
	(95%CI)	(95%CI)	(95%CI)	(95%CI)	(95%CI)
Mutil-tree XGBoost	0.985	0.934	0.938	0.943	0.929
	(0.982–0.987)	(0.924–0.944)	(0.928–0.949)	(0.933–0.952)	(0.918–0.940)
Simple-tree XGBoost	0.971	0.915	0.900	0.908	0.907
	(0.967–0.975)	(0.903–0.926)	(0.887–0.913)	(0.897–0.920)	(0.894–0.919)
Logistic-11	0.869	0.712	0.878	0.864	0.738
	(0.858–0.879)	(0.694–0.731)	(0.864–0.892)	(0.848–0.880)	(0.720–0.755)
Logistic-6	0.864	0.727	0.860	0.849	0.744
	(0.853–0.875)	(0.709–0.746)	(0.845–0.875)	(0.834–0.865)	(0.726–0.761)

AUC: area under the receiver operating characteristic curve; PPV: positive predictive value; NPV: negative predictive value; CI: Confidence Interval

## Discussion

A retrospective study of DC patients hospitalized in five third-level hospitals in southwest China showed that six characteristics, including TB, Na, ALB, PTA, WBC count and NLR were important predictors of the risk of infection in patients with DC. The simple-tree XGBoost model based on these six significant features shows good prediction performance. In training set, it had an AUROC of 0.971, sensitivity of 91.5%, specificity of 90.0%, PPV of 90.8%, and NPV of 90.7%.

More and more studies have confirmed that it is convenient and effective to use laboratory biological indicators to build prediction models. Wang et al. established a prognosis model by combining conventional laboratory indicators with COVID-19 patients. The model based on the combination of neutrophils, lymphocytes, platelets and IL-2R showed good performance in predicting the death of COVID-19 patients. When the critical value was 0.572, the sensitivity and specificity of the prediction model were 90.74% and 94.44%, respectively [31]. In a retrospective cohort study, the researchers used laboratory indicators such as hemoglobin, platelet count, white blood cell count, urea nitrogen, creatinine, glucose, sodium, potassium, and total bicarbonate to construct a multivariate LR model to predict in-hospital mortality of hospitalized patients. A good model calibration and fit were observed (Hosmer-Lemeshow = 13.9, P = 0.18) [32]. The simple-tree XGBoost model constructed in this study can also provide a simple screening tool for medical providers in the primary health care setting, so as to quickly identifying patients at high risk of infection in a single visit.

In a study aimed at constructing a multivariate predictive model for SBP in patients with liver cirrhosis, researchers found that blood neutrophil percentage was a significant predictor of SBP [33]. However, among the five indicators ultimately included in the prediction model, blood neutrophil percentage has the lowest importance compared to the other four indicators. Interestingly, in this study, NLR was the most important predictor for infection in DC patients, indicating that NLR’s sensitivity in predicting infection seems to be superior to blood neutrophil percentage. In addition, in this study, all six features included in the simple-tree XGBoost model have appeared in other studies on constructing prediction model for infection in patients with liver cirrhosis, indicating that the six features selected in this study have high clinical practicality in predicting infection [34–37].

PTA is a classic index used to judge the severity of liver disease [38]. Its sensitivity and specificity for various liver diseases are different in clinical evaluations, but a decrease in its level generally indicates that the liver function of the patients was damaged to different degrees. Llucia Tito et al. found that PTA was an independent predictor of liver cirrhosis complicated with SBP infection. In this study, a decreased PTA was found to be a risk factor for DC complicated with infection, and the risk of developing an infection would increase 0.04-fold when PTA decreased by 1% [39]. Hypoalbuminemia is also an independent risk factor for infection in DC patients. The low level of ALB reflects that the patient’s liver function and nutritional status are poor, the detoxification function of the body is reduced, and the ability to resist pathogenic bacteria is significantly reduced, which makes the patient prone to infection [40]. TB and Na were also proved to be poor predictors of infection [41, 42].

WBC count was another key predictor in the simple-tree XGBoost model. WBC count is an important component of the body’s defense system as a traditional indicator for detecting infectious diseases such as viruses and bacteria [43]. Autoimmune disease, infection or septicemia can cause excessive consumption of granulocytes, resulting in granulocytopenia. During the diagnosis of infected patients, the detection of patients’ WBC count can make a specific analysis of patients’ inflammation; However, in some patients with non bacterial infection, WBC count in patients will also show constant changes due to the influence of external environment [44, 45]. Cheng et al. found that WBC count was an important risk factor for nosocomial bacterial infection in COVID-19 patients in tertiary hospitals. It is worth noting that compared with WBC count [(4.0 ~ 10.0) × 10^9^/L], patients with WBC count (> 10.0 × 10^9^/L or ≤ 4.0 × 10^9^/L) have a 7.38 fold increased risk of nosocomial bacterial infection [46]. The study by Huang also demonstrated that WBC count (threshold > 10 × 10^9^/L) and procalcitonin to lactic acid ratio (threshold > 0.438) may help identify early stages of infection in patients with diabetic ketoacidosis, and combining these two markers may help with specificity [47].

NLR is a particularly interesting parameter. It is believed that liver cirrhosis has immune insufficiency, while neutrophils can reflect the immediate response of the body to inflammation, protect the body against bacterial infection [48–50], and lymphocyte level can reflect the immune level and nutritional status of the body. In patients with liver cirrhosis, the intestinal barrier is destroyed, intestinal flora changes, and pathogen-associated molecular patterns produced by bacteria, such as endotoxin, enter the blood circulation [51, 52]. Neutrophils can produce a large number of proinflammatory or anti-inflammatory cytokines, such as IL-6, IL-8, IL-17, when pathogen-associated molecular patterns and damage-associated molecular patterns are produced by liver cell necrosis. These cytokines in turn promote the activation of neutrophils [51]. In the process of disease development, patients often have lymphocytopenia, which may be related to the increase of lymphocyte apoptosis in the process of inflammation [53]. Therefore, NLR is an indicator that can reflect the overall immune status of the body. At the same time, a large number of studies have also confirmed that NLR can be used to evaluate the long-term or short-term prognosis of patients with stable or decompensated cirrhosis and cirrhosis with or without acute liver failure [48, 54–56].

In 2020, the annual per capita disposable income of rural households in China was approximately 17,132 yuan, which is approximately one-third of the income of urban households [57]. Financial cost may be the leading barrier to screen DC patients for the risk of infection. Because of immune response dysfunction, infection poses a huge risk to patients with DC and indicates the beginning of the terminal phase of this disease, but the known risk factors have not fully clarified this relationship. Thus, it is important to minimize the number of variables in diagnostic tools as much as possible in medically underserved settings. The population with limited access to infection care may benefit from our simple-tree XGBoost model, which was developed based on restricted medical resources and would not incur additional expenditures.

The advantage of this study is to use multicenter electronic medical record data to develop a infection prediction model. However, this study still has some limitations. First, due to retrospective research, the causal relationship between risk factors and infection should be carefully considered. Second, some important potential influencing factors were not included in this study because of significant data missing. Third, this study can only be regarded as a pilot study. More features and larger sample studies would be conducted to verify and improve the overall performance of the model in future.

## Conclusion

Our study suggests that a simple predictive model could provide added value as an automated screening tool to DC patients for infection. We identified six candidate features, including TB, Na, ALB, PTA, WBC count and NLR measured at hospital admission, as critical infection risk biomarkers for DC patients. The simple-tree XGBoost model conducted by the six significant features can help to predict infection of DC patients with accurately > 95% precision and > 95% sensitivity.

### Electronic supplementary material

Below is the link to the electronic supplementary material.

Supplementary Material 1

## Data Availability

The datasets used for this study are available on request to the corresponding author.

## References

[1] Angeli P, Bernardi M, Villanueva C, Francoz C, Mookerjee RP, Trebicka J (2018). EASL Clinical Practice Guidelines for the management of patients with decompensated cirrhosis. J Hepatol.

[2] D’Amico G, Morabito A, D’Amico M, Pasta L, Malizia G, Rebora P (2018). New concepts on the clinical course and stratification of compensated and decompensated cirrhosis. Hep Intl.

[3] Costentin CE, Layese R, Bourcier V, Cagnot C, Marcellin P, Guyader D (2018). Compliance with Hepatocellular Carcinoma Surveillance Guidelines Associated with increased lead-time adjusted survival of patients with compensated viral cirrhosis. Gastroenterology.

[4] Fleming KM, Aithal GP, Card TR, West J (2010). The rate of decompensation and clinical progression of disease in people with cirrhosis: a cohort study. Aliment Pharmacol Ther.

[5] Merwe SVd, Chokshi S, Bernsmeier C, Albillos A (2021). The multifactorial mechanisms of bacterial infection in decompensated cirrhosis. J Hepatol.

[6] Solà E, Solé C, Ginès P (2016). Management of uninfected and infected ascites in cirrhosis. Liver International: Official Journal of the International Association for the Study of the Liver.

[7] Gallo A, Dedionigi C, Civitelli C, Panzeri A, Corradi C, Squizzato A (2020). Optimal management of cirrhotic ascites: a review for internal medicine physicians. J Translational Intern Med.

[8] Reuken PA, Stallmach A, Bruns T (2013). Mortality after urinary tract infections in patients with advanced cirrhosis - relevance of acute kidney injury and comorbidities. Liver International: Official Journal of the International Association for the Study of the Liver.

[9] Marciano S, Dirchwolf M, Bermudez CS, Sobenko N, Haddad L, Ber FG (2018). Spontaneous bacteremia and spontaneous bacterial peritonitis share similar prognosis in patients with cirrhosis: a cohort study. Hep Intl.

[10] Benz F, Mohr R, Tacke F, Roderburg C. Pulmonary complications in patients with liver cirrhosis. 2020;8(3):150–8. 10.2478/jtim-2020-0024 PMC7534492 33062591

[11] Fernández J, Tandon P, Mensa J, Garcia-Tsao G (2016). Antibiotic prophylaxis in cirrhosis: good and bad. Hepatology (Baltimore MD).

[12] Yamaguchi D, Sakata Y, Yoshida H, Furukawa NE, Tsuruoka N, Higuchi T (2017). Effectiveness of endoscopic hemostasis with soft coagulation for Non-Variceal Upper gastrointestinal bleeding over a 12-Year period. Digestion.

[13] Alabsawy E, Shalimar, Sheikh MF, Ballester MP, Acharya SK, Agarwal B (2022). Overt hepatic encephalopathy is an independent risk factor for de novo infection in cirrhotic patients with acute decompensation. Aliment Pharmacol Ther.

[14] Toledo C, Salmerón JM, Rimola A, Navasa M, Arroyo V, Llach J (1993). Spontaneous bacterial peritonitis in cirrhosis: predictive factors of infection resolution and survival in patients treated with cefotaxime. Hepatology.

[15] Thuluvath PJ, Morss S, Thompson R (2001). Spontaneous bacterial peritonitis—in-hospital mortality, predictors of survival, and health care costs from 1988 to 1998. Am J Gastroenterol.

[16] Saberi-Karimian M, Khorasanchi Z, Ghazizadeh H, Tayefi M, Saffar S, Ferns GA (2021). Potential value and impact of data mining and machine learning in clinical diagnostics. Crit Rev Clin Lab Sci.

[17] Jayatilake SMDAC, Ganegoda GU (2021). Involvement of machine learning tools in Healthcare decision making. J Healthc Eng.

[18] Shu S, Ren J, Song J (2021). Clinical application of machine learning-based Artificial Intelligence in the diagnosis, prediction, and classification of Cardiovascular Diseases. Circulation Journal: Official Journal of the Japanese Circulation Society.

[19] Mangasarian OL, Wild EW (2006). Multisurface proximal support vector machine classification via generalized eigenvalues. IEEE Trans Pattern Anal Mach Intell.

[20] Cichy RM, Kaiser D (2019). Deep neural networks as scientific models. Trends Cogn Sci.

[21] Chen T, Guestrin C, XGBoost:. A Scalable Tree Boosting System. Proceedings of the 22nd acm sigkdd international conference on knowledge discovery and data mining (pp. 785–794). ACM. 10.1145/2939672.2939785.

[22] Steinmeyer C, Wiese L (2020). Sampling methods and feature selection for mortality prediction with neural networks. J Biomed Inform.

[23] Auret L, Aldrich C (2012). Interpretation of nonlinear relationships between process variables by use of random forests. Miner Eng.

[24] Bai Y, Bain M. Optimizing weighted lazy learning and Naive Bayes classification using differential evolution algorithm. J Ambient Intell Humaniz Comput. 2021(prepublish):1–20.

[25] Kim H-J, Han D, Kim J, Kim D, Ha B, Seog W (2020). An Easy-to-use machine learning model to predict the prognosis of patients with COVID-19: Retrospective Cohort Study. J Med Internet Res.

[26] Huang Y, Chen H, Zeng Y, Liu Z, Ma H, Liu J. Development and Validation of a Machine Learning Prognostic Model for Hepatocellular Carcinoma Recurrence After Surgical Resection&#13. Frontiers in Oncology. 2021;10:593741. 10.3389/fonc.2020.593741 PMC7882739 33598425

[27] Collins GS, Reitsma JB, Altman DG, Moons KGM (2015). Transparent reporting of a multivariable prediction model for individual prognosis or diagnosis (TRIPOD): the TRIPOD Statement. BMC Med.

[28] Campbell KA, Trivedi HD, Chopra S (2021). Infections in cirrhosis: a guide for the Clinician. Am J Med.

[29] Kulkarni AV, Premkumar M, Arab JP, Kumar K, Sharma M, Reddy N (2022). Early diagnosis and Prevention of Infections in cirrhosis. Semin Liver Dis.

[30] Guan X, Zhang B, Fu M, Li M, Yuan X, Zhu Y (2021). Clinical and inflammatory features based machine learning model for fatal risk prediction of hospitalized COVID-19 patients: results from a retrospective cohort study. Ann Med.

[31] Wang F, Hou H, Wang T, Luo Y, Tang G, Wu S (2020). Establishing a model for predicting the outcome of COVID-19 based on combination of laboratory tests. Travel Med Infect Dis.

[32] Blanco N, Leekha S, Magder L, Jackson SS, Tamma PD, Lemkin D (2020). Admission laboratory values accurately predict In-hospital mortality: a Retrospective Cohort Study. J Gen Intern Med.

[33] Tu B, Zhang YN, Bi JF, Xu Z, Zhao P, Shi L (2020). Multivariate predictive model for asymptomatic spontaneous bacterial peritonitis in patients with liver cirrhosis. World J Gastroenterol.

[34] Yang Q, Jiang XZ, Zhu YF, Lv FF (2020). Clinical risk factors and predictive tool of bacteremia in patients with cirrhosis. J Int Med Res.

[35] Hu Y, Chen R, Gao H, Lin H, Wang J, Wang X (2021). Explainable machine learning model for predicting spontaneous bacterial peritonitis in cirrhotic patients with ascites. Sci Rep.

[36] Huynh NC, Vo TD (2023). Validation of a new simple scoring system to predict spontaneous bacterial peritonitis in patients with cirrhosis and ascites. BMC Gastroenterol.

[37] Termsinsuk P, Auesomwang C (2020). Factors that predict recurrent spontaneous bacterial peritonitis in cirrhotic patients. Int J Clin Pract.

[38] Drolz A, Horvatits T, Roedl K, Rutter K, Staufer K, Kneidinger N (2016). Coagulation parameters and major bleeding in critically ill patients with cirrhosis. Hepatology (Baltimore MD).

[39] Titó L, Rimola A, Ginès P, Llach J, Arroyo V, Rodés J (1988). Recurrence of spontaneous bacterial peritonitis in cirrhosis: frequency and predictive factors. Hepatology (Baltimore MD).

[40] Trebicka J (2022). Role of albumin in the treatment of decompensated liver cirrhosis. Curr Opin Gastroenterol.

[41] Takahashi N, Nakada T-A, Walley KR, Russell JA (2021). Significance of lactate clearance in septic shock patients with high bilirubin levels. Sci Rep.

[42] Ismail MK, Daboul I, Waters B, Fleckenstein JF, Vera SR, Riely CA (2001). Liver transplastion for hepatic sarcoidosis: long term follow-up and recurrence after liver transplantion, a single center experience. Gastroenterology.

[43] Safuan SNM, Tomari MRM, Zakaria WNW (2018). White blood cell (WBC) counting analysis in blood smear images using various color segmentation methods. Measurement.

[44] Honda T, Uehara T, Matsumoto G, Arai S, Sugano M (2016). Neutrophil left shift and white blood cell count as markers of bacterial infection. Clin Chim Acta.

[45] Ishimine N, Honda T, Yoshizawa A, Kawasaki K, Sugano M, Kobayashi Y (2013). Combination of white blood cell count and left shift level real-timely reflects a course of bacterial infection. J Clin Lab Anal.

[46] Cheng K, He M, Shu Q, Wu M, Chen C, Xue Y (2020). Analysis of the risk factors for nosocomial bacterial infection in patients with COVID-19 in a Tertiary Hospital. Risk Manage Healthc Policy.

[47] Huang B, Yang S, Ye S. Systemic infection predictive value of procalcitonin to lactic acid ratio in diabetes ketoacidosis patients. Diabetes, metabolic syndrome and obesity: targets and therapy. 2022;15:2127–33. 10.2147/DMSO.S371437 PMC9325875 35911501

[48] Kalra A, Wedd JP, Bambha KM, Gralla J, Golden-Mason L, Collins C (2017). Neutrophil-to-lymphocyte ratio correlates with proinflammatory neutrophils and predicts death in low model for end-stage liver disease patients with cirrhosis. Liver transplantation: official publication of the American Association for the study of Liver Diseases and the International Liver. Transplantation Soc.

[49] Tritto G, Bechlis Z, Stadlbauer V, Davies N, Francés R, Shah N (2011). Evidence of neutrophil functional defect despite inflammation in stable cirrhosis. J Hepatol.

[50] Mookerjee RP, Stadlbauer V, Lidder S, Wright GAK, Hodges SJ, Davies NA (2007). Neutrophil dysfunction in alcoholic hepatitis superimposed on cirrhosis is reversible and predicts the outcome. Hepatology (Baltimore MD).

[51] Albillos A, Lario M, Álvarez-Mon M (2014). Cirrhosis-associated immune dysfunction: distinctive features and clinical relevance. J Hepatol.

[52] Kalaitzakis E (2014). Gastrointestinal dysfunction in liver cirrhosis. World J Gastroenterol.

[53] Viers BR, Thompson RH, Lohse CM, Cheville JC, Leibovich BC, Boorjian SA (2016). Pre-treatment neutrophil-to-lymphocyte ratio predicts tumor pathology in newly diagnosed renal tumors. World J Urol.

[54] Cai Y-J, Dong J-J, Dong J-Z, Chen Y, Lin Z, Song M (2017). A nomogram for predicting prognostic value of inflammatory response biomarkers in decompensated cirrhotic patients without acute-on-chronic liver failure. Aliment Pharmacol Ther.

[55] Liu H, Zhang H, Wan G, Sang Y, Chang Y, Wang X (2014). Neutrophil-lymphocyte ratio: a novel predictor for short-term prognosis in acute-on-chronic hepatitis B liver failure. J Viral Hepatitis.

[56] Zhang H, Sun Q, Mao W, Fan J, Ye B (2016). Neutrophil-to-lymphocyte ratio predicts early mortality in patients with HBV-Related decompensated cirrhosis. Gastroenterol Res Pract.

[57] China NBoSo (2021). China Statistical Yearbook 2021.

