# Supplementary material for: Clinical Data based XGBoost Algorithm for infection risk prediction of patients with decompensated cirrhosis: a 10-year (2012–2021) Multicenter Retrospective Case-control study
Source: BMC Gastroenterol. 2023 Sep 13;23:310. doi: 10.1186/s12876-023-02949-3 (PMC10500933; doi:10.1186/s12876-023-02949-3)
Supplement: Supplementary file 1 — Supplementary Material 1 [file 12876_2023_2949_MOESM1_ESM.docx]

**Supplementary information**

**Figure S1. Flow of inclusions and exclusions**


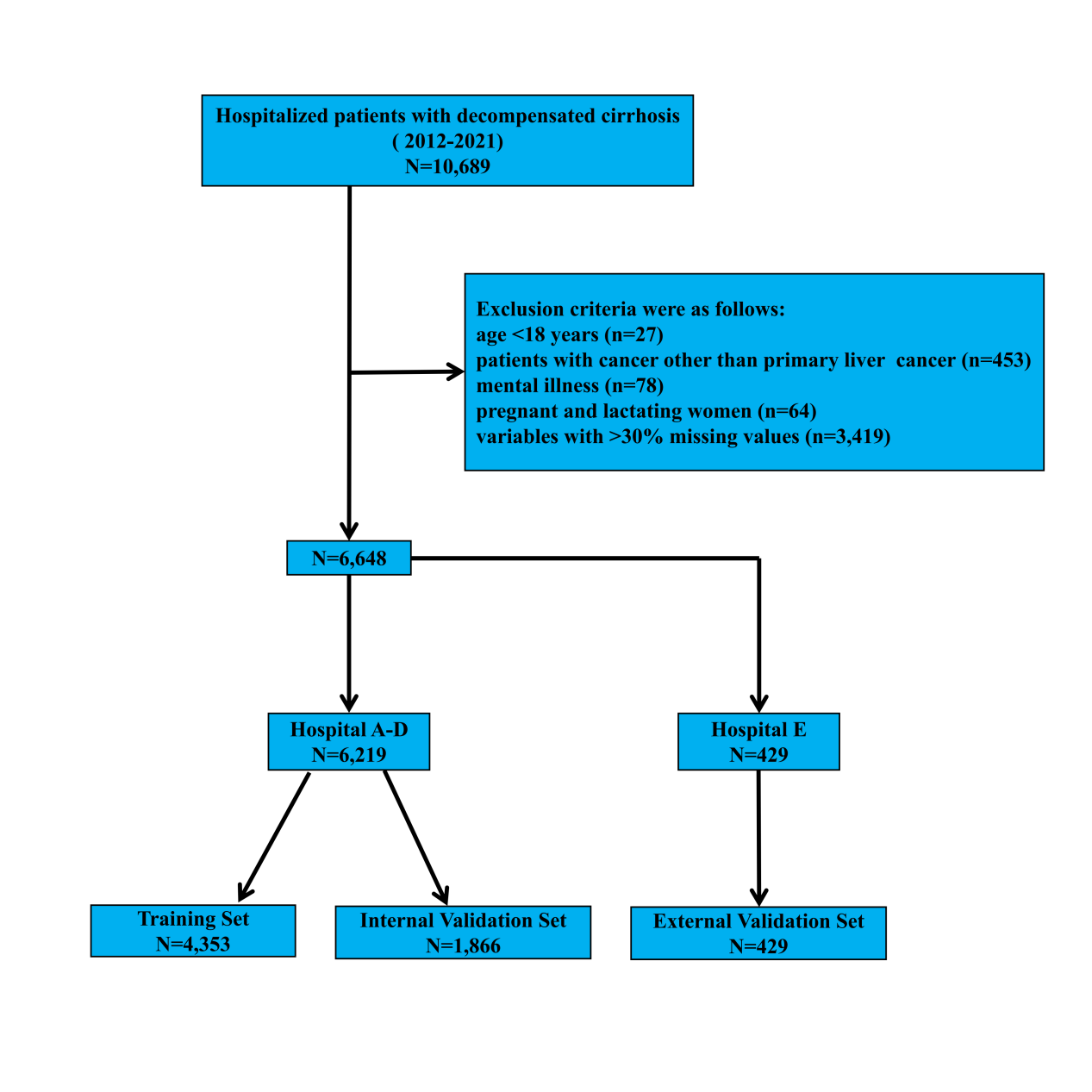


**
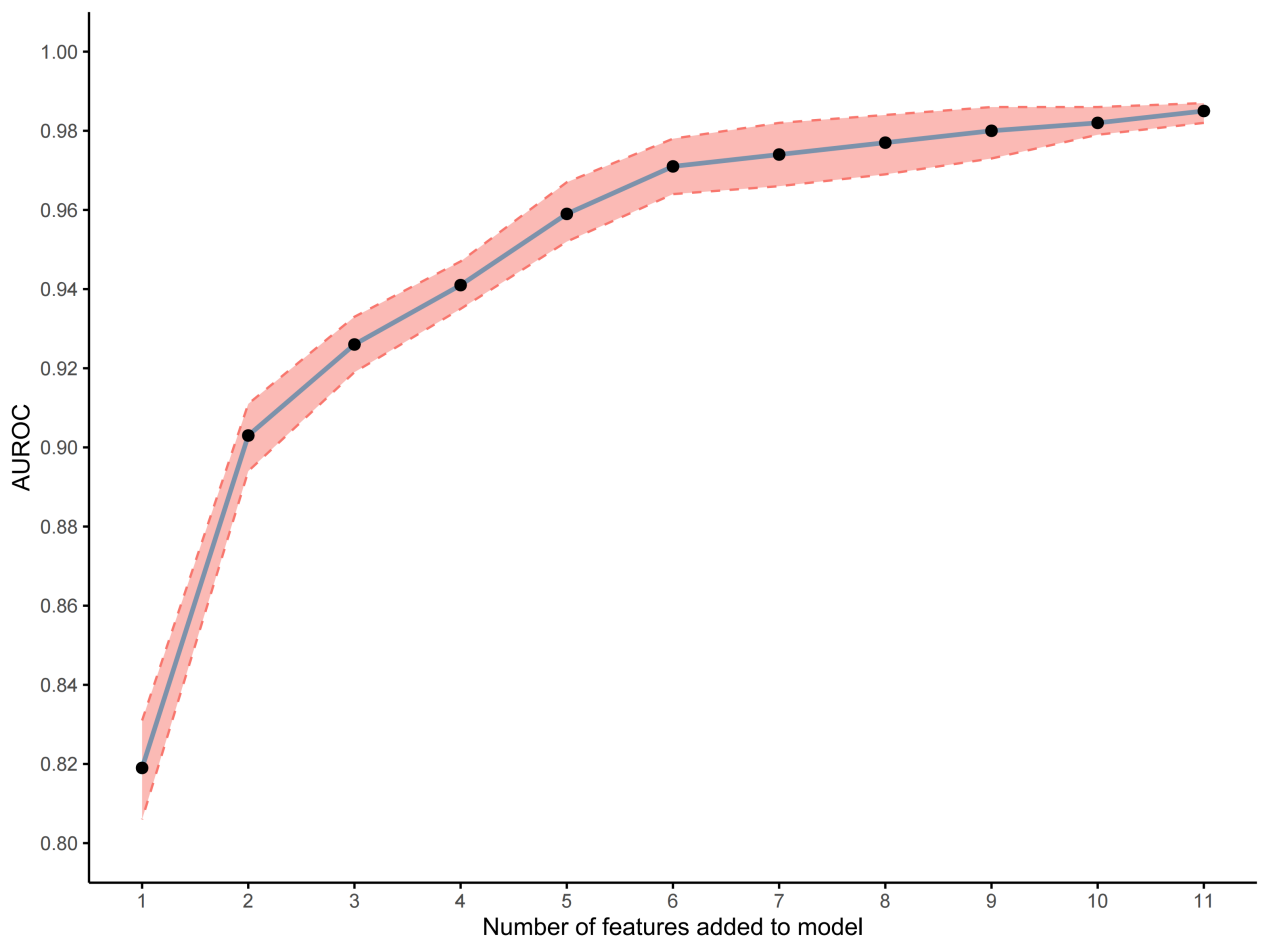
**

**Figure S2. Identification of the optimal variables numbers for a prediction of infection**

**Table S1. Comparison of continuous variables in the training and internal validation sets before and after multiple imputation**

| Variables | Before interpolation | After interpolation | *P* values |
| --- | --- | --- | --- |
| TP (IQR, g/L) | 64.89(58.60,71.60) | 64.70(58.60,71.60) | 0.815 |
| TB (IQR, umol/L) | 29.60(17.20,72.10) | 29.60(17.40,71.70) | 0.827 |
| Hemoglobin (IQR, g/L) | 109.00(87.00,127.00) | 108.00(85.00,127.00) | 0.130 |
| Na (IQR, mmol/L) | 139.30(136.20,141.70) | 139.30(136.20,141.70) | 0.755 |
| K (IQR, mmol/L) | 3.87(3.56,4.19) | 3.87(3.56,4.19) | 0.836 |
| ALB (IQR, g/L) | 31.20(27.20,35.40) | 31.20(27.20,35.40) | 0.905 |
| PTA (IQR, %) | 63.60(49.00,78.00) | 64.00(49.00,78.00) | 0.608 |
| BUN (IQR, mmol/L) | 5.43(4.16,7.40) | 5.40(4.12,7.39) | 0.547 |
| Cr (IQR, umol/L) | 67.20(56.40,81.90) | 67.00(56.00,81.70) | 0.290 |
| RBC count (IQR, ×10^9^/L) | 3.55(2.93,4.12) | 3.52(2.89,4.12) | 0.661 |
| WBC count (IQR, ×10^9^/L) | 4.40(3.07,6.50) | 4.30(3.00,6.34) | 0.815 |
| NLR (IQR) | 3.33(2.11,5.65) | 3.40(2.08,5.80) | 0.827 |

*TP: total protein; TB: total bilirubin; Na: blood sodium; K: blood potassium; ALB: albumin; PTA: prothrombin activity; BUN: blood urea nitrogen; Cr: creatinine; RBC: Red blood cell; WBC: white blood cell; NLR: neutrophils to lymphocytes ratio; IQR: interquartile range.*

**Table S2. Comparison of continuous variables in external validation set before and after multiple imputation**

| Variables | Before interpolation | After interpolation | *P* values |
| --- | --- | --- | --- |
| TP (IQR, g/L) | 64.59(57.68,70.70) | 64.30(57.60,70.60) | 0.838 |
| TB (IQR, umol/L) | 23.60(15.80,48.40) | 24.50(15.80,48.30) | 0.952 |
| Hemoglobin (IQR, g/L) | 99.00(78.00,123.00) | 99.00(78.00,123.00) | 0.929 |
| Na (IQR, mmol/L) | 138.20(135.85,140.22) | 138.30(135.90,140.30) | 0.695 |
| K (IQR, mmol/L) | 3.87(3.57,4.18) | 3.86(3.55,4.18) | 0.857 |
| ALB (IQR, g/L) | 32.75(28.3,38.18) | 32.70(28.20,37.80) | 0.814 |
| PTA (IQR, %) | 64.00(52.00,76.00) | 64.00(52.00,76.00) | 0.886 |
| BUN (IQR, mmol/L) | 5.57(4.13,7.93) | 5.45(4.14,7.79) | 0.825 |
| Cr (IQR, umol/L) | 64.60(54.18,75.53) | 65.00(54.20,75.60) | 0.907 |
| RBC count (IQR, ×10^9^/L) | 3.37(2.79,3.94) | 3.36(2.78,3.95) | 0.919 |
| WBC count (IQR, ×10^9^/L) | 4.16(2.70,5.79) | 4.13(2.69,5.77) | 0.870 |
| NLR (IQR) | 3.40(2.24,6.08) | 3.40(2.23,6.08) | 0.965 |

*TP: total protein; TB: total bilirubin; Na: blood sodium; K: blood potassium; ALB: albumin; PTA: prothrombin activity; BUN: blood urea nitrogen; Cr: creatinine; RBC: Red blood cell; WBC: white blood cell; NLR: neutrophils to lymphocytes ratio; IQR: interquartile range.*

**Table S3. The formula details of the performance criteria**

| Indexs | Calculation formula |
| --- | --- |
| sensitivity | Sensitivity=TP/(TP+FN) |
| specificity | Specificity=TN/(TN+FP) |
| PPV | PPV=TP/(TP+FP) |
| NPV | NPV=TN/(TN+FN) |

*PPV: positive predictive value; NPV: negative predictive value; TP:ture positive; TN: ture negative; FP: false positive; FN: false negative.*

**Figure S3. AUROC in internal validation set**

**
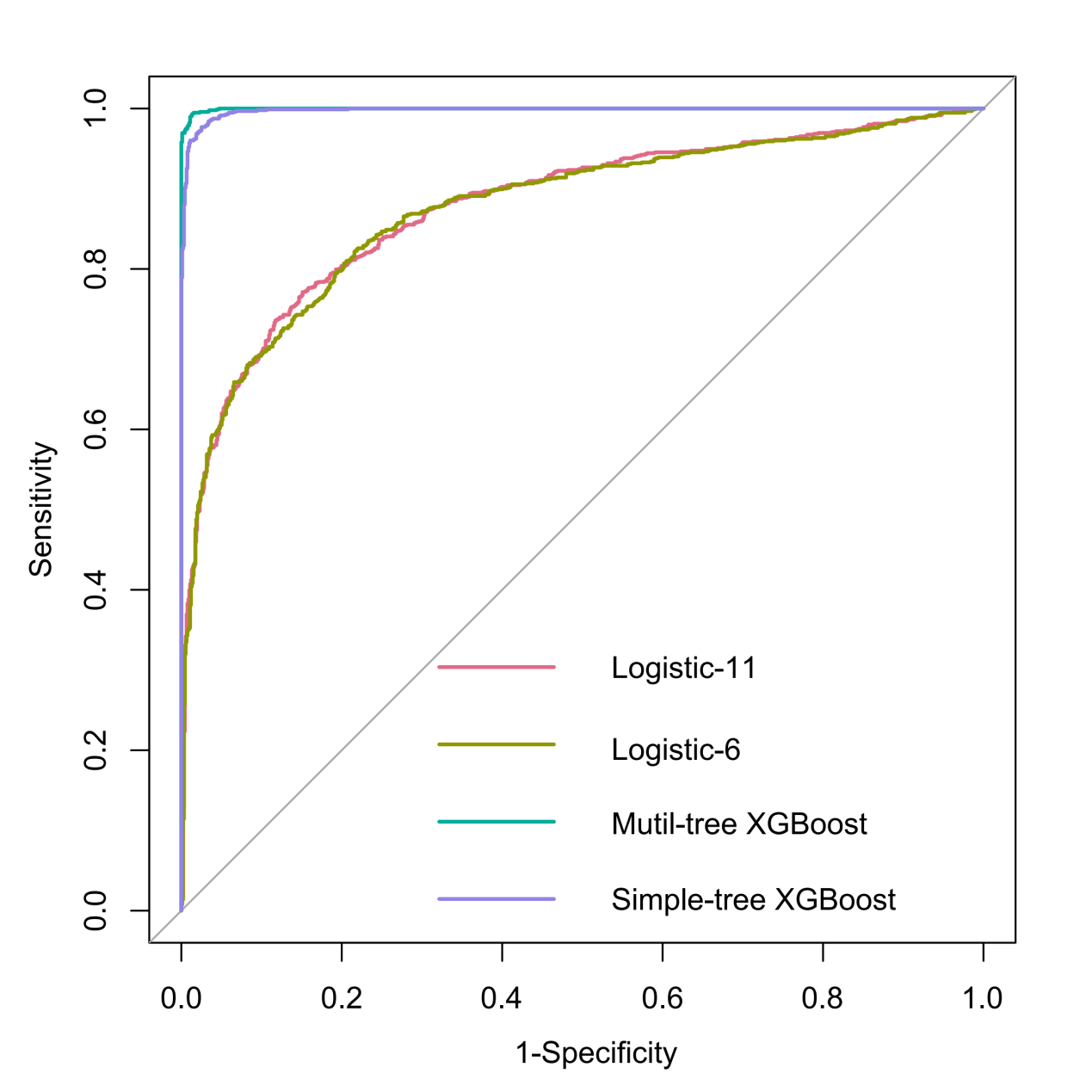
**

**Table S4. Detailed performance metrics for the four models in internal validation set**

| Models | AUROC | Sensitivity | Specificity | PPV | NPV |
| --- | --- | --- | --- | --- | --- |
|  | (95%CI) | (95%CI) | (95%CI) | (95%CI) | (95%CI) |
| Mutil-tree XGBoost | 1.000 | 0.993 | 0.987 | 0.987 | 0.992 |
|  | (0.999-1.000) | (0.987-0.998) | (0.979-0.994) | (0.980-0.995) | (0.987-0.998) |
| Simple-tree XGBoost | 0.998 | 0.977 | 0.975 | 0.976 | 0.976 |
|  | (0.996-0.999) | (0.967-0.986) | (0.965-0.985) | (0.966-0.986) | (0.966-0.986) |
| Logistic-11 | 0.878 | 0.771 | 0.849 | 0.842 | 0.780 |
|  | (0.862-0.894) | (0.745-0.798) | (0.826-0.872) | (0.818-0.866) | (0.755-0.806) |
| Logistic-6 | 0.875 | 0.822 | 0.784 | 0.799 | 0.808 |
|  | (0.859-0.891) | (0.797-0.846) | (0.758-0.811) | (0.774-0.824) | (0.782-0.834) |

*AUC: area under the receiver operating characteristic curve; PPV: positive predictive value; NPV: negative predictive value; CI: Confidence Interval.*

**Figure S4. AUROC in external validation set**

**
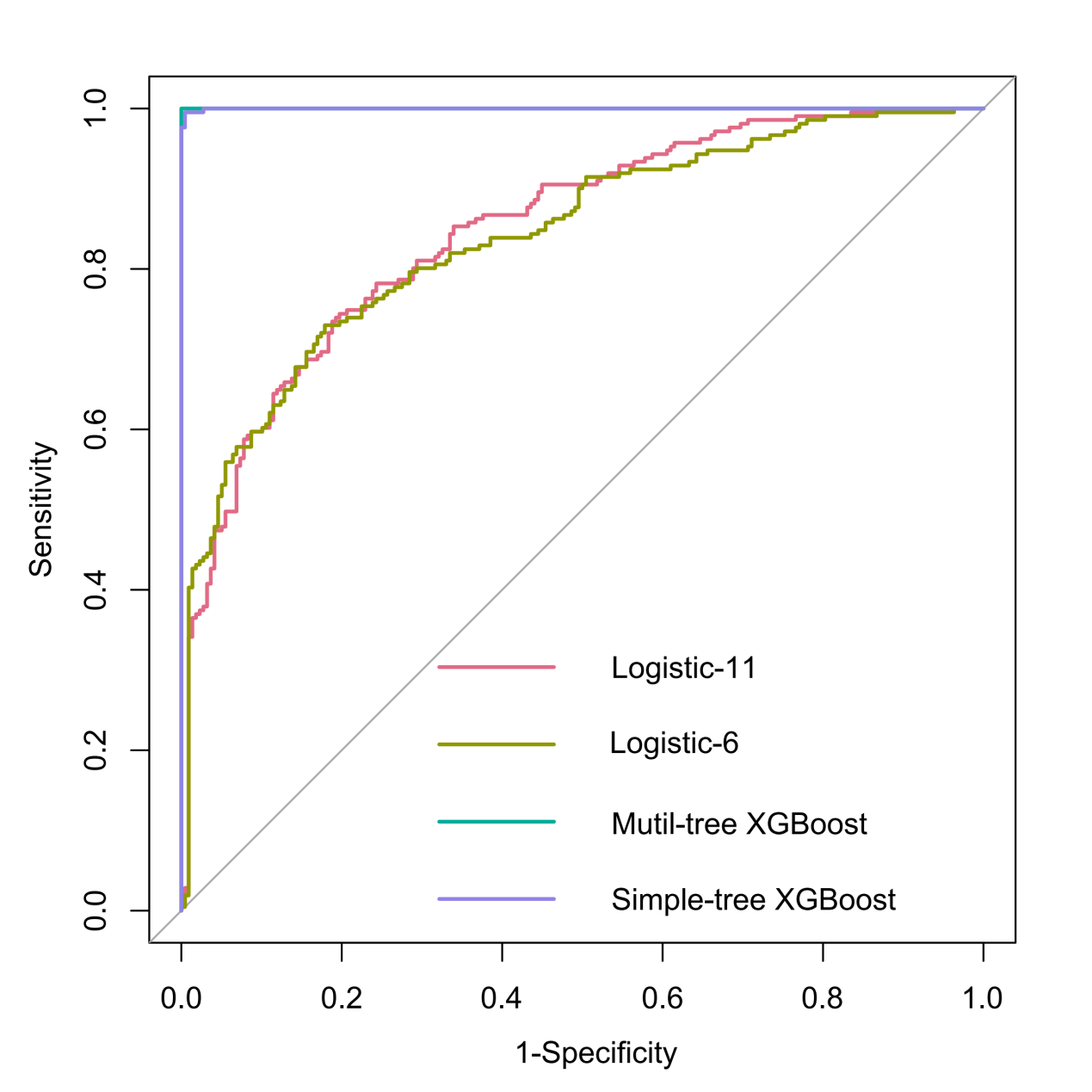
**

**Table S5. Detailed performance metrics for the four models in external validation set**

| Models | AUROC | Sensitivity | Specificity | PPV | NPV |
| --- | --- | --- | --- | --- | --- |
|  | (95%CI) | (95%CI) | (95%CI) | (95%CI) | (95%CI) |
| Mutil-tree XGBoost | 1.000 | 1.000 | 0.995 | 0.995 | 1.000 |
|  | (1.000-1.000) | (1.000-1.000) | (0.986-1.000) | (0.986-1.005) | (1.000-1.000) |
| Simple-tree XGBoost | 1.000 | 0.995 | 0.995 | 0.995 | 0.995 |
|  | (0.999-1.000) | (0.986-1.000) | (0.986-1.000) | (0.986-1.005) | (0.986-1.004) |
| Logistic-11 | 0.849 | 0.744 | 0.803 | 0.785 | 0.764 |
|  | (0.813-0.884) | (0.685-0.803) | (0.750-0.856) | (0.728-0.842) | (0.709-0.819) |
| Logistic-6 | 0.840 | 0.730 | 0.821 | 0.798 | 0.758 |
|  | (0.803-0.877) | (0.670-0.790) | (0.770-0.872) | (0.741-0.855) | (0.704-0.813) |

*AUC: area under the receiver operating characteristic curve; PPV: positive predictive value; NPV: negative predictive value; CI: Confidence Interval.*

**Table S6. Detailed performance metrics in different centers**

| Centers | Sensitivity | Specificity | PPV | NPV | AUROC | P values |
| --- | --- | --- | --- | --- | --- | --- |
|  | (95%CI) | (95%CI) | (95%CI) | (95%CI) | (95%CI) |  |
| Total | 0.915 | 0.900 | 0.908 | 0.907 | 0.971 | / |
|  | (0.903-0.926) | (0.887-0.913) | (0.897-0.920) | (0.894-0.919) | (0.967-0.975) |  |
| A | 0.888 | 0.930 | 0.933 | 0.883 | 0.970 | 0.871 |
|  | (0.874-0.902) | (0.918-0.942) | (0.922-0.945) | (0.868-0.897) | (0.966-0.975) |  |
| B | 0.903 | 0.942 | 0.938 | 0.909 | 0.976 | 0.355 |
|  | (0.868-0.938) | (0.914-0.969) | (0.909-0.967) | (0.875-0.942) | (0.966-0.986) |  |
| C | 0.784 | 0.969 | 0.967 | 0.795 | 0.948 | 0.335 |
|  | (0.651-0.916) | (0.908-1.000) | (0.902-1.031) | (0.668-0.922) | (0.904-0.993) |  |
| D | 0.929 | 0.939 | 0.929 | 0.939 | 0.974 | 0.857 |
|  | (0.833-1.000) | (0.858-1.000) | (0.833-1.024) | (0.858-1.021) | (0.938-1.000) |  |

*AUC: area under the receiver operating characteristic curve; PPV: positive predictive value; NPV: negative predictive value; CI: Confidence Interval.*
